# A New Look at the Most Successful Prodrugs for Active Vitamin D (D Hormone): Alfacalcidol and Doxercalciferol

**DOI:** 10.3390/molecules14103869

**Published:** 2009-09-29

**Authors:** Noboru Kubodera

**Affiliations:** Chugai Pharmaceutical Co., Ltd., 2-1-1, Nihonbashi-Muromachi, Chuo-ku, Tokyo 103-8324, Japan; E-Mail: kuboderanbr@chugai-pharm.co.jp; Tel.: +81 3 3273 1010; Fax: +81 3 3281 0191

**Keywords:** prodrugs, vitamin D, D hormone, alfacalcidol, doxercalciferol, practical syntheses

## Abstract

Alfacalcidol (1α-hydroxyvitamin D_3_) has been widely used since 1981 as a prodrug for calcitriol (1α,25-dihydroxyvitamin D_3_) in the treatment of hypocalcemia, chronic renal failure, hypoparathyroidism and osteoporosis. More recently, doxercalciferol (1α-hydroxyvitamin D_2_) has been used since 1999 as a prodrug for 1α,25-dihydroxyvitamin D_2_ for the treatment of secondary hyperparathyroidism. Currently, six forms of vitamin D are known. They range from vitamin D_2_ to vitamin D_7_ and are distinguished by their differing side chains. Only vitamin D_2_ and vitamin D_3_ have been found to be biologically active based on the elucidation of activation pathways. Alfacalcidol and osteoporosis/doxercalciferol and secondary hyperparathyroidism are discussed, with a new look at old compounds including their practical syntheses.

## 1. Introduction [1,2]

**Scheme 1:**
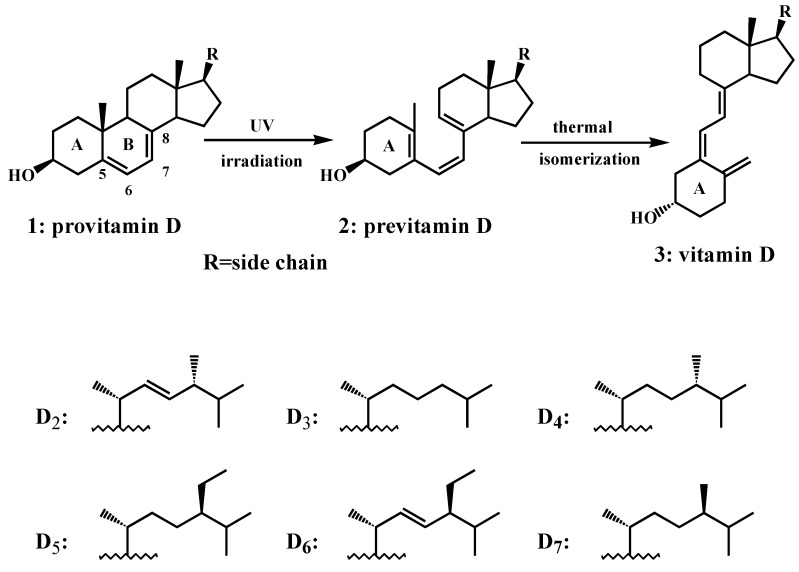
Biosynthesis of vitamin D (**3**) from provitamin D (**1**) *via* previtamin D (**2**) and side chain structure of vitamin D_2_ through vitamin D_7_.

Rickets, a bone disease, has been common since ancient times, particularly in Europe. A therapeutic factor that is known to be a contaminant in *oleum morrhuae*, was initially thought to be vitamin A, however, subsequent studies found that this substance was different from vitamin A and it was named vitamin D. The code ‘D’ was derived from the fact that it was found next to vitamin C. Currently, six forms of vitamin D are known. They range from vitamin D_2_ to vitamin D_7_ and they are distinguished by their different side chains. Although there are reasonable grounds for why vitamin D_1_ is missing, these are not discussed here. The formation of all types of vitamin D involves previtamin D as a common precursor. Cleavage of the B-ring in provitamin D (**1**) takes place upon exposure of the 5,7-diene sterol (provitamin D) (**1**) to ultraviolet light, followed by subsequent thermal isomerization of previtamin D (**2**) to produce vitamin D (**3**), the secosteroid. As the A-ring of vitamin D inverts from the original steroid framework during thermal isomerization, α and β designations which show the configuration of substituents of the A-ring of vitamin D are now opposite from the normal convention (according to the IUPAC nomenclature) (Scheme 1).

Of the six forms of vitamin D, vitamin D_2_ through vitamin D_7_, only vitamin D_2_ and vitamin D_3_ have been found to be biologically active based on the elucidation of activation pathways. Accordingly, the term vitamin D usually refers to vitamin D_2_ and/or vitamin D_3_. Vitamin D_3_, cholecalciferol, is derived from provitamin D (7-dehydrocholesterol) on the skin following ultraviolet irradiation by sunlight, or taken in by eating seafood, among other foods. Vitamin D_2_, ergocalciferol, is derived from provitamin D (ergosterol) and also taken internally by consuming fungi such as Japanese mushrooms. In the US, it is added to milk. While partially synthesized in the skin, oral ingestion is essential for vitamin D_2_ and vitamin D_3_ intake, which are referred to as native vitamin D. Within this context, substance ‘D’ is definitely positioned as a vitamin. Vitamin D, which it is ingested from external sources or synthesized in the skin, is metabolized to 25-hydroxyvitamin D following 25-hydroxylation of the side chain in the liver. Since this hydroxylation in the liver is not strictly controlled (contrary to hydroxylation at the 1α-position in the kidney), 25-hydroxyvitamin D serves as a vitamin D reserve in the body that is stably circulating in the blood for a long time while binding to the specific vitamin D binding protein (DBP) [3]. Hence 25-hydroxyvitamin D concentration in the blood represents vitamin D sufficiency. It is interesting to note that the promiscuous manner of 25-hydroxylation reaction in the body utilizes two of the most successful prodrugs and medications, alfacalcidol and doxercalciferol as described below. 

**Scheme 2:**
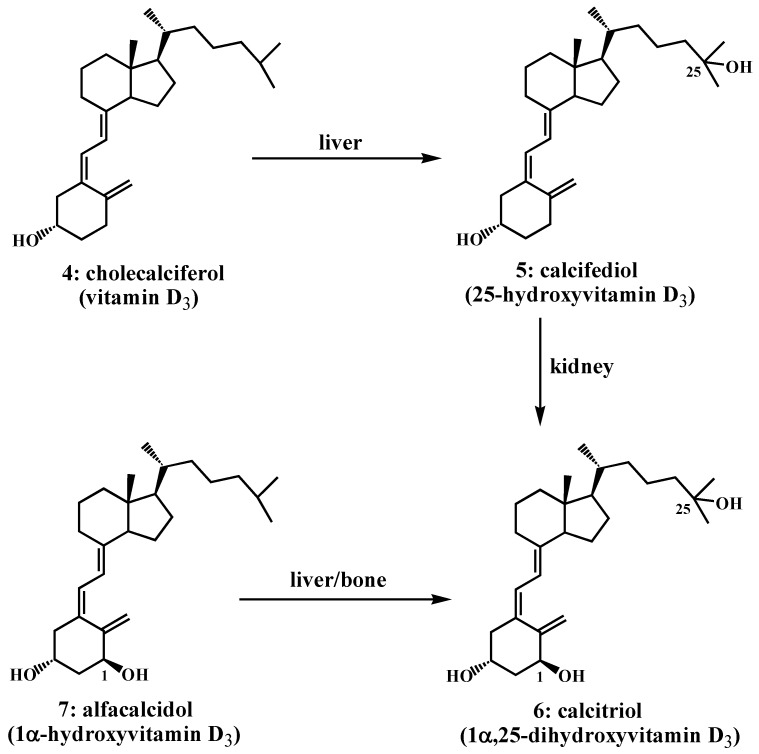
Activation of vitamin D (cholecalciferol; vitamin D_3_) (**4**) and prodrug (alfacalcidol; 1α-hydroxyvitamin D_3_) (**7**) to D hormone (calcitriol; 1α,25-dihydroxyvitamin D_3_) (**6**).

25-Hydroxyvitamin D is absorbed, as needed, into proximal renal tubules from the lumen by megalin, hydroxylated at the 1α-position by 1α-hydroxylase, and ultimately metabolized to 1α,25-dihydroxyvitamin D [4]. The metabolites are delivered as active vitamin D to targeted organs such as the small intestine, bone, kidney and parathyroid gland which enables various biological responses after binding to vitamin D receptors (VDR) [5]. Initially, active vitamin D was recognized as a substance involved in calcium metabolism. The main actions, which include absorption of calcium from the small intestine, mobilization of bone mineral, and reabsorption of calcium by kidney, serve to increase the concentration of calcium in the blood and, are needed to successfully maintain the body’s calcium level. Studies have revealed that VDR exists in tissues and organs all over the body, such as skin, brain, and muscle, and is not limited to the small intestine, kidney, bone, and parathyroid gland. This suggests that vitamin D deficiency might be associated with various diseases. In contrast to 25-hydroxylation in the liver, 1α-hydroxylation in the kidney is strictly controlled by parathyroid hormone (PTH) or fibroblast growth factor-23 (FGF-23). Hence, 1α,25-dihydroxyvitamin D is referred to as active vitamin D. In this context, substance ‘D’ is completely positioned as a hormone, which is why 1α,25-dihydroxyvitamin D is referred to as D hormone. Scheme 2 illustrates the activation pathway of vitamin D_3_ (cholecalciferol) (**4**) to 1α,25-dihydroxyvitamin D_3_ (calcitriol) (**6**) *via* 25-hydroxyvitamin D_3_ (calcifediol) (**5**) (Scheme 2).

## 2. Alfacalcidol

### 2.1. Development of the prodrug alfacalcidol

Patients with renal damage who require artificial kidney dialysis are expected to develop vitamin D deficiency due to a disorder of vitamin D activation caused by insufficient hydroxylation in the kidney. An effective treatment for such individuals with renal impairment is through chemical treatment, specifically the administration of a vitamin D derivative possessing a hydroxyl group at 1α-position. As part of these efforts, 1α-hydroxyvitamin D_3_ (generic name: alfacalcidol) was developed in Japan by Chugai Pharmaceutical Co., Ltd., and the Teijin Institute for Bio-Medical Research in 1981 as the first prodrug of calcitriol for medical use under the sales names of Alfarol (Chugai) and Onealfa (Teijin). As described above, hydroxylation in the liver is comparatively promiscuous, and the prodrug (alfacalcidol, **7**) is activated to the active form (calcitriol, **6**) in the body. In addition, it has been revealed that this transformation may also take place in the bone, but to a lesser degree (Scheme 2). 

A key question is why the prodrug alfacalcidol was developed as a pharmaceutical product, rather than the exact final substance calcitriol. There are two reasons for this: one is that it was difficult to develop calcitriol for medical use due to issues associated with patent rights; the other - discussed in detail in the subsequent chapter- is that alfacalcidol had some advantages with respect to the cost of an industrial synthesis. Specifically, inexpensive and readily available cholesterol could be used as the starting material because alfacalcidol does not have a hydroxyl moiety at the 25-position of the side chain. Although the initial indications of alfacalcidol in 1981 included vitamin D deficiency and hypocalcemia, osteoporosis was added to the list of indications in 1983. Clinically, alfacalcidol has been the first-line treatment for osteoporosis in Japan for more than 25 years [6,7]. 

Osteoporosis is a disease caused by bone resorption overtaking bone formation and the creation of an imbalance between the two processes that is normally due to aging. Women in particular develop the disease more frequently than men because their bone mineral density is rapidly reduced after menopause. For the prevention of osteoporosis, it is very important to ingest enough calcium and get exposure to sunlight in order to form sufficient vitamin D from an early age. It is definitely a myth, however, that the administration of calcium and vitamin D is effective for the treatment of fractures in patients with osteoporosis. The administration of high-dose calcium would only be a burden on these advanced-age patients because the VDR level in their small intestine is low. Furthermore, if high-dose vitamin D was administered to them, sufficient D hormone would not be supplied because 25-hydroxyvitamin D would not be activated due to reduced renal hydroxylase activities. The author and colleagues recently found that the administration of high-dose vitamin D only increase bone strength to a certain level in ovariectomized rats (OVX rats), while prodrug for D hormone, alfacalcidol, treatment improves both strength and density dose dependently [8]. An explanation for such a phenomenon has not yet been elucidated. It is suggested, however, that only D hormone calcitriol is produced from prodrug alfacalcidol for blood circulation, while a number of minor metabolites are produced in addition to D hormone from vitamin D. Almost no roles for these metabolites have been clarified. It should be acknowledged that the physiological need of ingesting vitamin D is significantly different from the administration of D hormone for the treatment of osteoporosis. 

In clinical use, the intestinal calcium absorption effect of alfacalcidol becomes active in a daily dosage of 0.25 μg to 0.5 μg and becomes saturated with higher dose. At dose levels between 0.75 μg to 1 μg, alfacalcidol suppresses PTH and inhibits bone resorption in adults who ingest a normal amount of calcium. These findings suggest a therapeutic threshold for alfacalcidol, wherein bone is intensified. On the other hand, bone resorption becomes encouraged at a dosage of 1.5 μg or more, which is the threshold to develop adverse effects. It has been pointed out that D hormone has a narrow therapeutic window. The author and colleagues recently demonstrated that D hormone promoted bone formation within the therapeutic threshold in parathyroidectomized rats, not due to intestinal calcium absorption or PTH suppression but due to its direct effect on bone [8]. We have found a new active vitamin D derivative, eldecalcitol [1α,25-dihydroxy-2β-(3-hydroxypropoxy)vitamin D_3_, developing code: ED-71], which possesses a hydroxypropoxy substituent at the 2-position of the A-ring of calcitriol. Eldecalcitol promotes potent bone formation in lower doses than alfacalcidol and has a wider window than alfacalcidol between therapeutic and bone resorption. It is therefore expected to be a useful therapy for osteoporosis. Please refer to the relevant study for details of eldecalcitol [9,10,11,12,13].

In Europe and the US, bisphosphonates (e. g., sodium alendronate, sodium risedronate, sodium ibandronate) or selective estrogen receptor modulators (SERM), such as raloxifene, have been mainly used for the treatment of osteoporosis. This is in contrast to Japanese clinical practice, which focuses on D hormone therapy [14]. Various reasons for this have been elaborated which include differences in the history of osteoporosis therapy development, the amount of calcium intake, the ratio of responders to non-responders due to gene polymorphism of VDR, and cultural differences. Of these reasons, the most significant is that in Western countries, the average amount of calcium intake is higher than in Japan, and vitamin D is added to milk and milk products. Hence, in Western countries, vitamin D as a treatment might induce hypercalcemia rather than have therapeutic effects on bone [15]. Currently SERM and bisphosphonates are also gaining ground as an accepted form of therapy in Japan.

Our recent pharmacokinetics and autoradiography studies using tritiated alfacalcidol or tritiated calcitriol administered to rats revealed the disparate character of alfacalcidol compared to calcitriol. Plasma concentration of transformed active vitamin D_3_ after oral or intravenous administration of tritiated alfacalcidol showed longer plasma half-life, lower maximum concentration, and lower area under the curve than those after treatment of tritiated calcitriol. Furthermore, active vitamin D_3_ fraction in rat bone after alfacalcidol treatment was sustained for longer than that after calcitriol treatment [16]. We also confirmed in the microautoradiography studies that localization of radioactivity in bone after treatment with triatiated alfacalcidol or tritiated calcitriol is observed in osteoblast nuclei. Radioactivity of active vitamin D_3_ in the nuclei after alfacalcidol treatment was also sustained longer than that after calcitriol treatment [17]. Although these results might suggest a beneficial therapeutic utility of prodrug (alfacalcidol) over treatment of the active form of vitamin D_3_ (calcitriol), further basic and clinical studies are necessary to clarify the differences in the detailed characteristics between alfacalcidol and calcitriol.

### 2.2. Practical synthesis of alfacalcidol

**Scheme 3:**
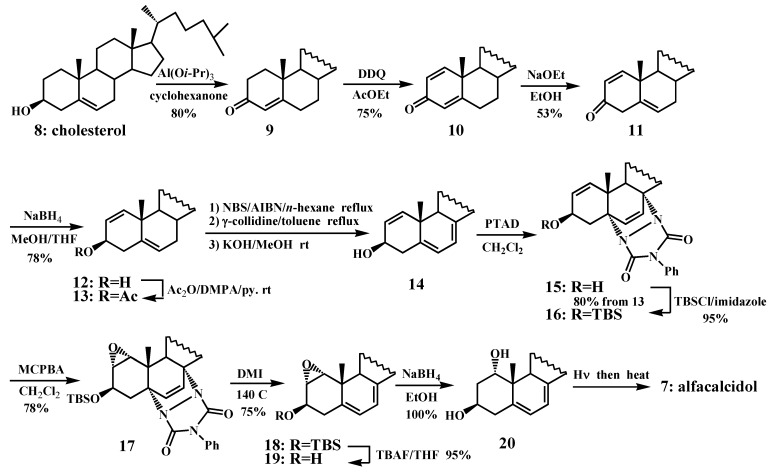
An example of the improved synthesis of alfacalcidol (**7**) from cholesterol (**8**).

Scheme 3 shows an example of the improved practical synthesis of alfacalcidol [18]. The synthetic features are:

1) Inexpensive and readily available cholesterol (**8**) is used as the starting material. If 25-hydroxylated cholesterol is used, basically a similar reaction to give calcitriol would follow, but with much higher costs than alfacalcidol.
2) 1α-Hydroxylation, which is done in the kidney, is replaced by stereoselective formation of an α-epoxide and subsequent regioselective epoxide cleavage by hydride reduction.
3) Introducing a biomimetic method, i.e., ultraviolet irradiation and thermal isomerization used to obtain the same reaction in the skin caused by sunlight following the synthesis of the 5,7-diene segment.

Thus, cholesterol (**8**) was oxidized with aluminum isopropoxide [Al(O*i*-Pr)_3_] in 80% yield to the 4-en-3-one **9**, which was further oxidized with 2,3-dichloro-5,6-dicyano-1,4-benzoquinone (DDQ) to 1,4-dien-3-one **10** in 75% yield. Treatment of **10** with sodium ethoxide (NaOEt) gave 1,5-diene-3-one **11** in 53% yield, which was reduced with sodium borohydride (NaBH_4_) yielding 3β-hydroxy-1,5-diene **12** in 78% yield. After protection of hydroxyl moiety in **12** as the acetate **13**, the 5,7-diene system in **14**, required for ultraviolet irradiation, was introduced by bromination with *N*-bromosuccinimide (NBS)/2,2’-azobisisobutyronitrile (AIBN) in hexane and dehydrobromination with γ-collidine in toluene after deacetylation. The 5,7-diene moiety in **14** was protected by forming an adduct with 4-phenyl-1,2,4-triazoline-3,5-dione (PTAD) to give the PTAD adduct **15** in 80% yield from **13**. The hydroxyl group in **15** was protected as the *t*-butyldimethylsilyl (TBS) ether **16** in 95% yield, which was then regio- and stereoselectively epoxidized with *m*-chloroperbenzoic acid (MCPBA) to give 1,2*a*-epoxide **17** in 78% yield. Retro-cycloaddition of the PTAD adduct **17** to regenerate the 5,7-diene system in **18** was carried out by simply heating (140 °C) **17** in 1,3-dimethyl-2-imidazolidinone (DMI) in 75% yield [19]. The 3β-hydroxyl moiety in **19**, obtained by deprotection of TBS group in **18** with tetrabutylammonium fluoride (TBAF), contributed to the regio- and stereoselective cleavage of epoxide with NaBH_4_ to give diol **20**, quantitatively. Finally, diol **20** was subjected to photolysis and thermal isomerization to afford alfacalcidol (**7**) (Scheme 3). The yields for converting steroidal frameworks (provitamin D) to secosteroids (previtamin D) by photolysis and thermal isomerization are usually moderate to low, since several structural byproducts such as lumisterol, tachisterol, etc. are formed, in addition to the requisite previtamin D [13].

## 3. Doxercalciferol

### 3.1. Development of the prodrug doxercalciferol

In 1981, basic research found the existence of VDR in various organs and tissues of the body and that D hormone facilitates differentiation-inducing and antiproliferartion activities in tumor cells. Prior to those findings D hormone had been considered to be involved in only calcium metabolism [21]. During that same year, alfacalcidol came onto the market. Following alfacalcidol treatment in patients with vitamin D deficiency or hypocalcemia, there was an increasing number of those with good clinical prognosis of concurrent rheumatoid arthritis or intractable dermatologic psoriasis vulgaris. It was also found that if the dose of alfacalcidol was increased to raise the therapeutic effect on comorbidities, hypercalcemia developed as an adverse effect before achieving the intended effect. Although specific reasons for the clinical effectiveness of D hormone for psoriasis vulgaris and rheumatoid arthritis were unknown, an increasing number of scientists associated the effect with the differentiation-inducing and antiproliferation activities found in basic research. Subsequently, many exploratory studies on synthesizing D hormone derivatives with indication of differentiation-inducing activity *in vitro* and calcemic activity *in vivo* were performed all over the world [22,23]. Doxercalciferol, 1α-hydroxyvitamin D_2_, is such a derivative showing potent PTH suppression properties with less calcemic activity than alfacalcidol [24].

Secondary hyperparathyroidism (2HPT) is a common disorder in patients with chronic renal failure. The pathogenesis of this disorder, characterized by increased PTH secretion and parathyroid gland hyperplasia, is attributed primarily to the retention of phosphate and the decreased capacity to produce active vitamin D. Low serum 1α,25-dihydroxyvitamin D reduces intestinal calcium transport and bone calcium mobilization, and high serum phosphate further contributes to the hypocalcemia by decreasing the free serum calcium. The parathyroid glands respond initially to the low calcium levels by increasing PTH secretion and synthesis, but prolonged hypocalcemia leads to parathyroid gland hyperplasia. Hyperphosphatemia can directly increase PTH synthesis, stabilize PTH mRNA, promote parathyroid hyperplasia, and decrease the calcium receptor. The fall in serum 1α,25-dihydroxyvitamin D reduces the direct suppressive effects of this hormone on PTH gene transcription and parathyroid cell hyperplasia and may contribute to the reduced levels of receptors for calcium and active vitamin D.

Prevention and treatment of 2HPT requires both the control of serum phosphate and restoration of 1α,25-dihydroxyvitamin D levels. Phosphate is commonly controlled by reducing dietary phosphate absorption, usually by oral administration of calcium-based phosphate binders. Replacement therapy with calcitriol or its prodrug alfacalcidol has been successfully used, but is often precluded by the narrow therapeutic window for suppression of PTH without hypercalcemia. For this reason, derivatives of vitamin D that retain the suppressive effect on PTH secretion but have lower calcemic and phosphatemic activity could provide a safer and more effective means of controlling 2HPT [25].

**Scheme 4:**
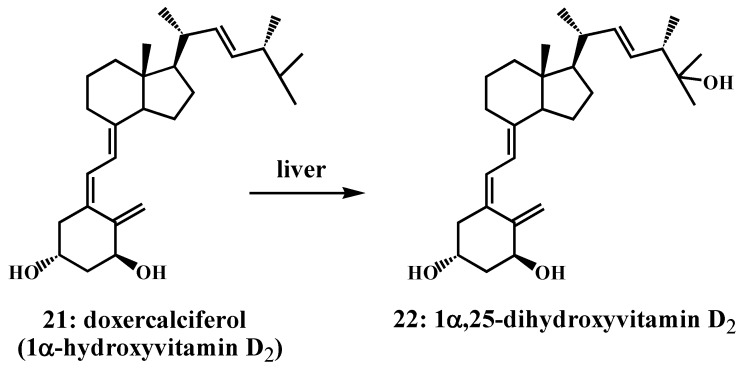
Conversion of prodrug (doxercalciferol: 1α-hydroxyvitamin D_2_, **21**) to D hormone (1α,25-dyhydroxyvitamin D_2_, **22**).

Doxercalciferol (**21**), developed by Bone Care International, was launched in 1999 with the sales name of Hectorol for the treatment of 2HPT in the US. Although doxercalciferol (**21**) is a prodrug of active vitamin D_2_ (1α,25-dihydroxyvitamin D_2_ **22**) (Scheme 4) and, like calcitriol (1α,25-dihydroxyvitamin D_3_), which is the counterpart of alfacalcidol, must be activated *in vivo*, the mechanism for the biological action of doxercalciferol is much less well understood than for alfacalcidol [26,27,28,29,30,31,32]. The results of a trial with doxercalciferol in renal failure patients with moderate to severe 2HPT (intact PTH greater than 400 pg/mL) were reported [33]. After an 8-week washout period, oral doxercalciferol was administered initially at a dose of 4 μg/day or 4 μg thrice weekly, and the dose was adjusted to maintain serum intact PTH levels between 130 and 250 pg/mL. The target goal was reached by 21 of the 24 patients. Mean serum calcium rose from 8.8 to 9.5 mg/dL during the 12-week study, but average phosphate levels did not change. Oral doxercalciferol was judged to be safe and effective in treating 2HPT and was thus approved for use in patients in the US. Intravenous doxercalciferol has also been introduced.

### 3.2. Practical synthesis of doxercalciferol

**Scheme 5:**
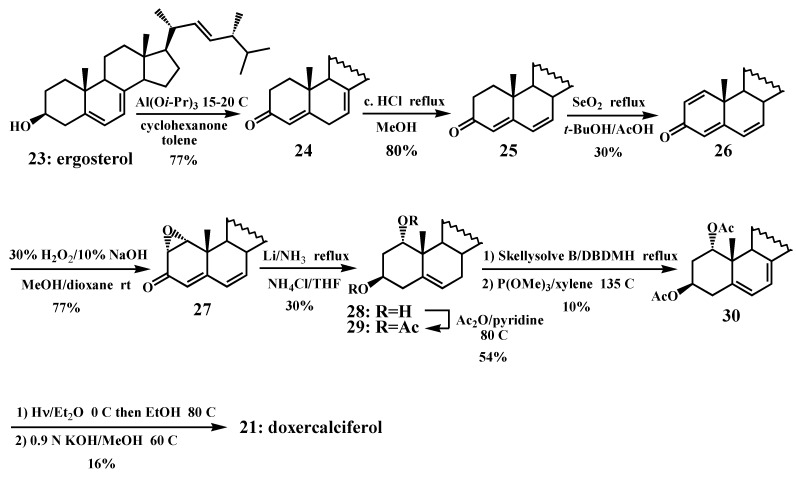
Practical synthesis of doxercalciferol (**21**) from ergosterol (**23**).

The ten-step synthetic route to doxercalciferol (**21**) starting from ergosterol (**23**) is well documented in the literature by DeLuca [34]. Thus, Oppenauer oxidation of ergosterol (**23**) gave 4,7,22-triene-3-one **24** in 77% yield, which was followed by acid catalyzed double bond conjugation according to the procedure outlined by Shepherd [35]. This sequence afforded 4,6,22-triene-3-one **25** in 80% yield. Selenium oxide (SeO_2_) oxidation of **25** led to 1,4,6,22-tetraene-3-one **26** in 30% yield. Upon treatment of **26** with alkaline hydrogen peroxide (H_2_O_2_), the α-epoxide **27** was obtained in 77% yield. Reduction of **27** with lithium-ammonium chloride (Li-NH_4_Cl) in liquid ammonia-tetrahydrofuran (NH_3_-THF) furnished, after chromatography and crystallization, a 30% yield of diol **28**, which, *via* its diacetate **29** (54% yield), was converted to the required 5,7-diene **30** by bromination with *N,N’*-dibromo-5,5-dimethylhydantoin (DBDMH)-dehydrobromination with trimethyl phosphate (P(OMe)_3_). Irradiation of **30** by a high-pressure mercury lamp followed by thermal isomerization in refluxing ethanol (EtOH) followed by diacetate hydrolysis gave doxercalciferol (**21**) in 16% yield [Scheme 5].

## 4. Conclusions and Future Prospects

As discussed, alfacalcidol for the treatment of osteoporosis and doxercalciferol for secondary hyperparathyroidism are two of the most successful examples of prodrugs that contribute to current clinical practice. However, since the original research began, vitamin D studies by scientists, including the author, seem to have paid too much attention to 1α,25-dihydroxyvitamin D, based on the recognition of calcitriol and 1α,25-dihydroxyvitamin D_2_ as the biologically active metabolites. In the body, there are many metabolites of vitamin D and whose biological roles are yet unknown. They may have physiological actions that maintain homeostasis. Over the past several years, research data suggesting an association of vitamin D with various diseases, such as cancer, diabetes and Alzheimer’s disease, have been reported. The author believes that the various findings accumulated through the developmental activities and clinical uses of alfacalcidol and doxercalciferol will likely lead to greater in-depth molecular understanding and to the development of an array of new drugs in this field [36].
